# Molecular characterization of *Cryptosporidium* spp. in dogs and cats in the city of Rio de Janeiro, Brazil, reveals potentially zoonotic species and genotype

**DOI:** 10.1371/journal.pone.0255087

**Published:** 2021-08-03

**Authors:** Amanda Gleyce Lima de Oliveira, Adriana Pittella Sudré, Teresa Cristina Bergamo do Bomfim, Helena Lúcia Carneiro Santos

**Affiliations:** 1 Universidade Federal Rural do Rio de Janeiro–Departamento de Parasitologia–Instituto de Veterinária–Instituto de Veterinária, Seropédica, Rio de Janeiro, Brazil; 2 Universidade Federal Fluminense–Departamento de Microbiologia e Parasitologia, Instituto Biomédico, Niterói, Rio de Janeiro, Brazil; 3 Instituto Oswaldo Cruz—Laboratório de Estudos Integrados em Protozoologia, Rio de Janeiro, Brazil; Guru Angad Dev Veterinary and Animal Sciences University, INDIA

## Abstract

Intestinal cryptosporidiosis is a diarrheal disease caused by protists of genus *Cryptosporidium* that infect a wide variety of hosts, primarily vertebrates. Due to the close contact between humans and their companion animals, especially dogs and cats, there is concern about the potential for zoonotic transmission of this enteric protozoan parasite by infected animals. This study aimed to perform a microscopic and molecular diagnosis of *Cryptosporidium* spp. in fecal samples from domiciled dogs and cats. One hundred and nineteen fecal samples were processed using sugar centrifugal flotation followed by molecular detection of *Cryptosporidium* spp. DNA using nested PCR. Subtyping of isolates positive for *C*. *parvum* was performed by sequence analysis of the 60 kDa glycoprotein gene (GP60). *Cryptosporidium* oocysts were detected in 7.8% (5/64) and 5.4% (3/55) of the fecal samples from dogs and cats, respectively. *Cryptosporidium canis* (n = 3) and *C*. *parvum* (n = 2) were the main species found in dogs, whereas *C*. *felis* (n = 3) was prevalent in cats. Subtype IIaA17G2R2 (potentially zoonotic) was identified in samples positive for *C*. *parvum*. Despite the low prevalence of *Cryptosporidium* observed in the domiciled dogs and cats, the presence of potentially zoonotic *C*. *parvum* in dogs evidences a public health concern. Further research is needed to better understand the epidemiology, source, and potential impacts of *Cryptosporidium* infection in cats and dogs.

## Introduction

Companion animals, especially cats and dogs, contribute significantly not only to the physical, social and emotional development of their owners, but also to facilitating recovery from certain diseases [1]. Most Brazilian homes have companion animals, especially cats and dogs [2], and Brazil has the second largest pet market [3] in the world.

Cat and dog owners are often unaware whether their pets host microorganisms of zoonotic potential [4]. Enteric cryptosporidiosis stands out among the possible zoonoses that humans can acquire through the contact with these animals [5, 6]. This disease is a public health concern, as its infectious form, the oocyst, is easily dispersed in the environment, presenting various fecal-oral infection routes, such as direct contact with infected humans (person-to-person transmission) or animals (zoonotic transmission), or indirectly through ingestion of contaminated food (foodborne transmission) and water (waterborne transmission) [6, 7].

Molecular studies have shown that most infections in dogs and cats are caused by *C*. *canis* and *C*. *felis*, respectively [8–11], indicating specificity for these hosts. However, *C*. *parvum* is not species specific and presents an array of hosts, including cattle, humans and, occasionally, dogs and cats [6, 12–14]. *Cryptosporidium parvum* is classified as the second species (after *C*. *hominis*) of genus *Cryptosporidium* most commonly diagnosed in humans, followed by *C*. *felis* and *C*. *canis* [15].

Several studies conducted in Brazil have detected *Cryptosporidium* spp. in dogs and cats, but they did not identify the species [16–35]. However, using molecular tools, *C*. *canis* and *C*. *felis* were diagnosed in sheltered dogs and cats, respectively, in Brazil [36]. *Cyptosporidium canis* and *C*. *parvum* have been detected in dog feces, and *C*. *felis* and *C*. *parvum* in cats, however subtyping of *C*. *parvum* was not performed [14].

Microscopic methods, which use morphological characteristics, are useful for clinical diagnosis; however, they are not able to distinguish between species, genotypes and subtypes of *Cryptosporidium* [8, 10]. This information is of fundamental importance to identify the sources of infection [37].

Among the subtyping techniques developed for *C*. *parvum*, DNA sequence analysis of the 60-kDa glycoprotein gene (GP60) is the most commonly used [38]. Through molecular characterization, it is possible to epidemiologically assess this parasite and establish prevention measures to control outbreaks of infection [39, 40].

This study was aimed to investigate the occurrence of *Cryptosporidium* in domestic cats and dogs, and to identify the species of *Cryptosporidium* and subtype of *Cryptosporidium parvum*.

## Materials and methods

### Region of collection and number of samples

**Fig 1:**
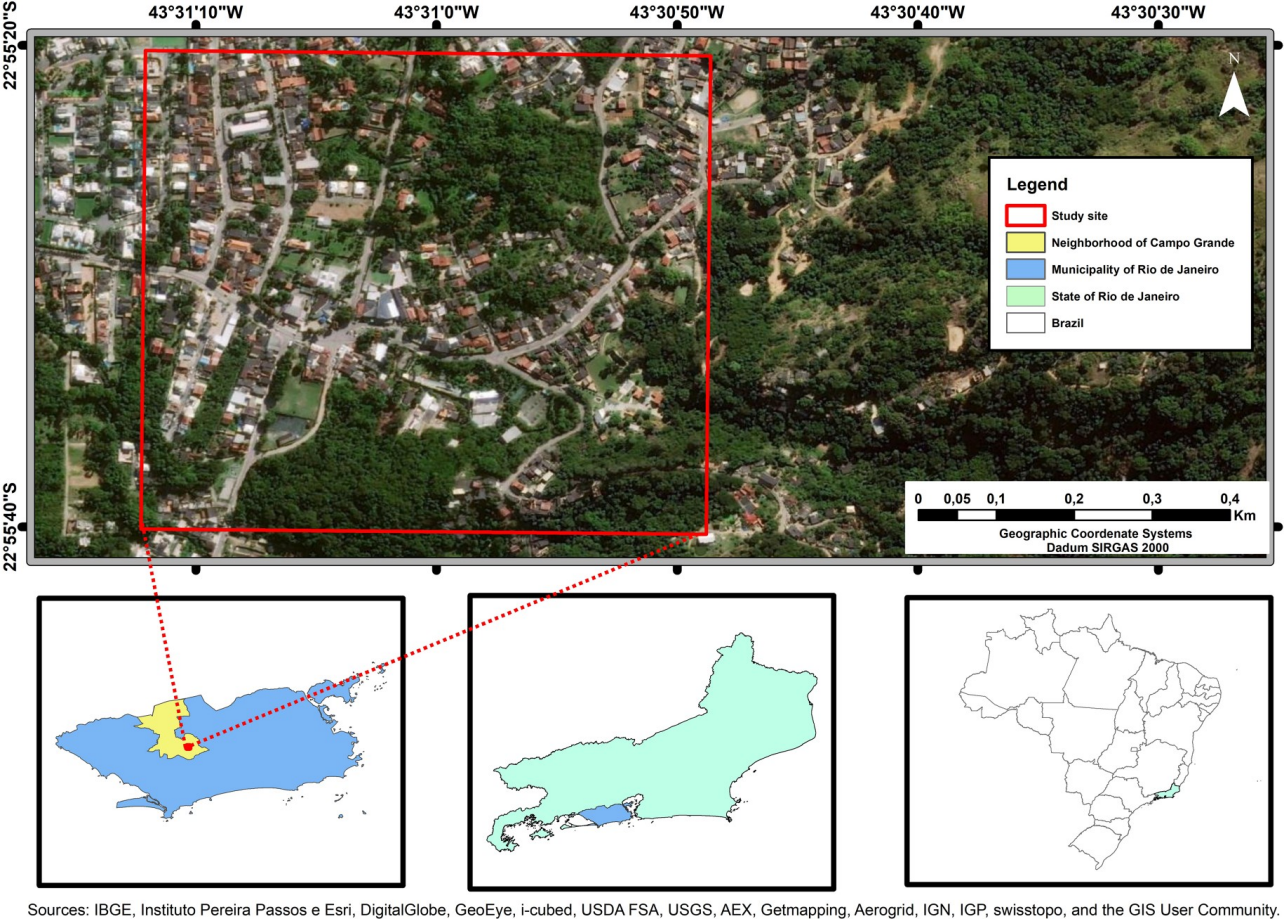
Geographical location of the study region where fecal samples from dogs and cats were collected.

Fecal samples from 119 companion animals, 64 dogs (*Canis lupus familiaris*) and 55 cats (*Felis catus*), were collected in the domiciles of their owners (Fig 1). Most of the 114 pet owners who agreed to participate in the study had only one companion animal, four domiciles had two animals, and only one domicile had three animals. These dogs and cats were fed animal feed, which often was not suitable for their age group, as well as leftovers. Many of these animals did not have access to veterinary assistance or good quality water.

This is a peri-urban region with presence of domestic (chickens and ducks) and free-living (pigeons and doves) birds, as well as of small livestock activities (raising of cattle, goats and pigs) for family subsistence. In addition, some owners have reported the presence of synanthropic rodents in this region. The vast majority of the dogs and cats investigated in this study had access to the surroundings of their domiciles. The fecal samples were collected randomly between September 2018 and June 2019.

Dog fecal samples were collected immediately after defecation from the superficial portion of the stools without contact with the soil, whereas the cat fecal samples were collected directly from the litter boxes, except for one household where there were three young cats with diarrhea. In this case, each cat was separately placed in a previously sanitized cage and the feces were collected immediately after defecation. For both dogs and cats, the fecal samples were placed in individual, screw-cap, sterile containers, identified, kept under refrigeration, and sent to the laboratory for processing.

The study sample was composed of dogs and cats of both sexes, aged 3 months to 13 years, with or without symptoms of diarrhea. The companion animals were classified into three age groups: young (≤1 year), adult (>1 and <7 years), and elderly (≥7 years) (Table 1).

**Table 1:** Percentage information on sex, age, and presence or absence of diarrhea in the population of dogs and cats (n = 119) in the neighborhood of Campo Grande, Rio de Janeiro, Brazil.

ANIMAL	SEX		AGE			DIARRHEA	
	Female	Male	≤ 1 year	> 1 and < 7 years	≥ 7 years	Yes	No
**Dogs (n = 64) (%)**	25 (39)	39 (61)	6 (11)	35 (54)	23 (36)	24 (37.5)	40 (62.5)
**Cats (n = 55) (%)**	20 (36.4)	35 (63.6)	9 (16.3)	32 (58.2)	14 (25.5)	17 (31)	38 (69)

### Ethics statement

This study was conducted according to the norms established by the Brazilian Council for the Control of Animal Experimentation (CONCEA) and was approved by the Ethics Committee on Animal Use of the Veterinary College of the Federal Rural University of Rio de Janeiro (UFRRJ) under protocol no. 070/2014.

### Laboratory procedures

#### Parasitological diagnosis

All fecal samples collected were subjected to the centrifugal flotation in saturated sugar solution technique [41], with modifications as follows: 10 g of each fecal sample were homogenized with 30 mL distilled water and filtered through disposable tamises. Subsequently, the material was placed in two conical 15 mL tubes and centrifuged (402.4 xg) for 10 min. Next, the supernatant was discarded, and one of the tubes containing sediment was stored in a freezer, while saturated sugar solution (1.30 g/mL specific density) was added to the other, which was then homogenized and centrifuged (402.4 xg) for 5 min. After that, saturated sugar solution was added to the surface of this tube, forming a meniscus on which a cover slip was placed and kept for 3 min. The cover slip was then mounted on a glass slide and examined under a microscope. The samples positive for *Cryptosporidium* spp. through observation of oocysts were subjected to molecular diagnosis.

#### Molecular diagnosis

*DNA extraction*. Total DNA was extracted from *Cryptosporidium* spp. positive fecal samples using a commercial kit (QIAamp^®^ Fast DNA Stool Mini Kit, Qiagen) following the manufacturer’s recommendations, but with two modifications: use of an 800-rpm stirrer with temperature control and elution of the samples in 100 μL buffer solution at the end of extraction.

*Cryptosporidium species identification using nested PCR*. Polymerase chain reaction (PCR) was carried out in two steps, and the 18S rRNA locus was used in both. In the first step, PCR products were sequenced in both directions using the amplification primers 18SF: 5`- TTC TAG AGC TAA TAC ATG CG-3`(forward) and 18SR: 5`- CCC ATT TCC TTC GAA ACA GGA-3`(reverse), and the master mix contained 4 mM MgCl_2_ (Invitrogen), 0.2 μM of each primer (18SF and 18SR) (Invitrogen), 1X Taq buffer (Invitrogen), 200 μM (each) deoxyribonucleotide triphosphate (dNTP) (Invitrogen), 1.0 U Platinum Taq Polymerase (Invitrogen), 2 μL of DNA, and ultrapure water (nuclease-free water) (Promega) until the final volume of 25 μL was reached. In the second step (Nested-PCR), the following primers were used: 18SNF: 5`- GGA AGG GTT GTA TTT ATT AGA TAA AG-3`(forward) and 18SNR: 5`- AAG GAG TAA GGA ACA ACC TCC A-3`(reverse), amplifying a fragment of 826–864 bp depending on the species. Nested PCR targeting 18S rRNA was performed [42, 43].

The same concentrations of the master mix reagents were used in this step with the following modifications: 2 mM MgCl_2_, 0.2 μM of the 18SNF primer, 0.2 μM of the 18SNR primer, and 1 μL of the amplicon.

Thermocycling conditions for the primary and secondary reactions were initial denaturation of 94°C for 3 min, followed by a total of 35 cycles [at 94°C for 45 s, at 58°C (primary PCR) and 59°C (nested PCR) for 45 s, and at 72°C for 1 min], and a final extension phase at 72°C for 7 min.

*Cryptosporidium baileyi* (KY710765) was used as positive control in the reactions for both cats and dogs. Dog and cat samples collected in three alternate days that presented negative results in the microscopic diagnosis and the PCR were used as negative control. A dog negative sample was used in the reactions for the subtyping of *Cryptosporidium parvum*, as previously described.

*Cryptosporidium parvum subtyping*. The positive samples for *C*. *parvum* were subtyped by PCR-sequence analysis of the GP60 gene. The AL3531 (5’-ATAGTCTCCGCTGTATTC-3’) and AL3535 (5’-GGAAGGAACGATGTATCT-3’) primers were used in the primary PCR and the AL3532 (5’-TCCGCTGTATTCTCAGCC-3’) and AL3534 (5’-GCAGAACCAGCATC-3’) primers were used in the nested-PCR to amplify a ~840-bp fragment [44].

The DNA amplification protocol for the target GP60 gene was similar to that previously described for the 18S rRNA gene, except for the primers and the annealing temperature, which was 56.8°C for both the primary and nested-PCRs.

The nested-PCR products were sequenced to determine *C*. *parvum* subtype families and their subtypes, which were named using the established GP60 subtype nomenclature [38, 45, 46].

### Sequencing and phylogenetic analysis

The amplified samples (18S and GP60) were purified using ExoSAP-IT^®^ PCR clean up reagent (USB; Cleveland, OH, USA) and sequenced by applying the nested-PCR primers in both directions using BigDye^®^ Terminator v3.1 cycle sequencing kits (Applied Biosystems) according to the manufacturer’s recommendations.

Analysis of the chromatograms and editing of the sequences were conducted utilizing the SeqMan Pro software (DNASTAR Inc., Madison, WI, USA). In addition, DNA sequences of *Cryptosporidium* spp. were obtained from GenBank, and multiple sequence alignment was performed using the ClustalW algorithm of the MEGA 6.0 software [47].

The consensus sequences were compared with previously published sequences using the BLASTn software from the NCBI server (http://www.ncbi.nlm.nih.gov/BLAST). Multiple sequence alignment was performed using the MAFFT online service (https://mafft.cbrc.jp/alignment/server/) [48]. Statistical selection of the best-fit model of nucleotide substitution in the SSU-rDNA gene was performed according to the ModelFinder method [49] based on the Bayesian Information Criterion (BIC) and Maximum Likelihood (ML) in IQ-TREE web server [50] and Bayesian inference analysis carried out using MrBayes v3.1.2 software [51]. The phylogenetic tree was constructed using the probabilistic Maximum Likelihood (ML) method, which is based on the GTR + G + I substitution model.

## Results

**Fig 2:**
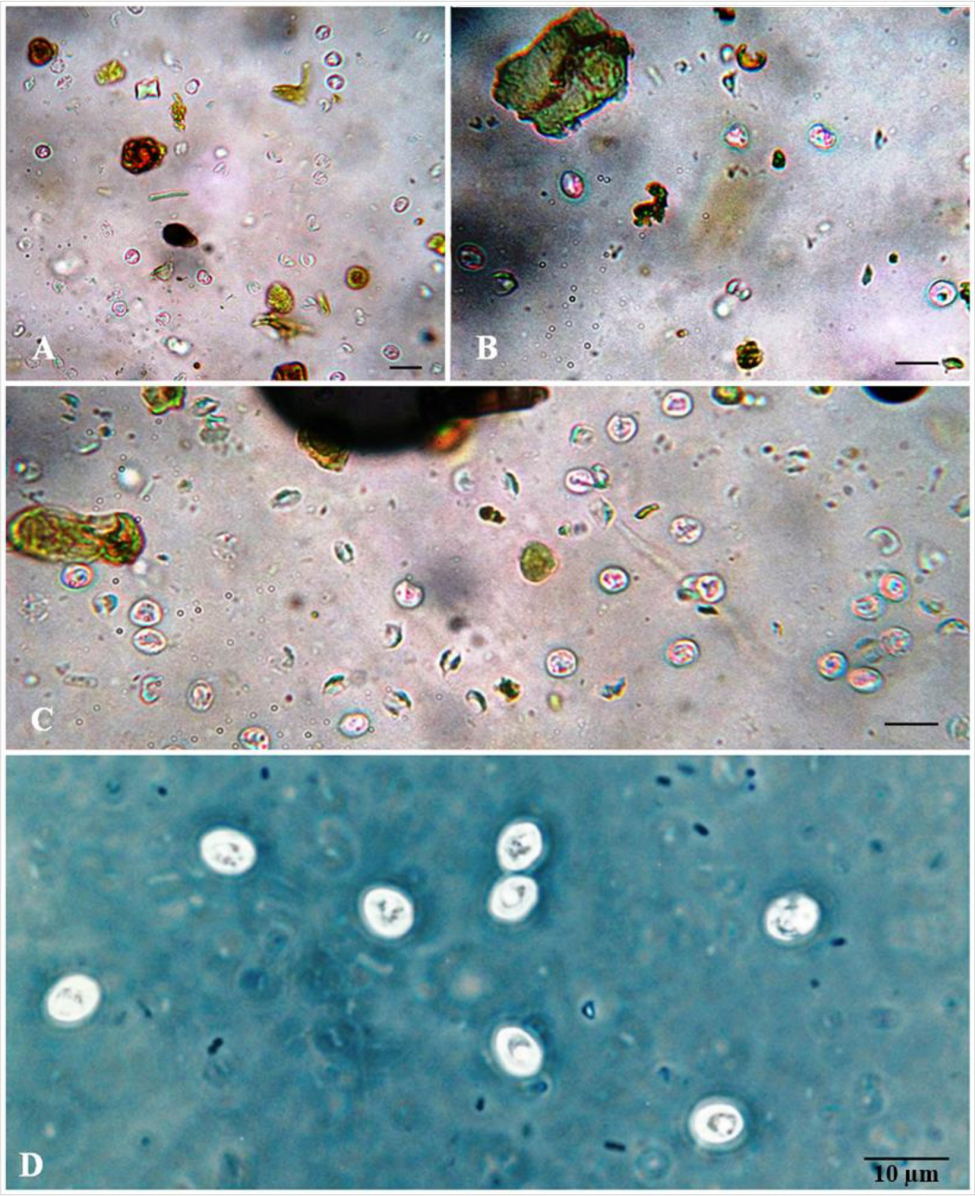
*Cryptosporidium* spp. oocysts from fecal samples of dogs and cats in the Campo Grande neighborhood, Rio de Janeiro, Brazil. A, B, and C: oocysts observed under bright-field microscope; D: oocysts observed under phase-contrast microscope.

Of the 109 companion animals analyzed, *Cryptosporidium* spp. oocysts (Fig 2) were detected in the fecal samples from five dogs (7.8%) and three cats (5.4%). Table 2 shows the positivity percentages of *Cryptosporidium* spp., as well as of other gastrointestinal parasites.

**Table 2:** Prevalence of gastrointestinal infections in fecal samples from 119 dogs and cats in the neighborhood of Campo Grande, Rio de Janeiro, Brazil.

PARASITES	Dogs (n = 64)		Cats (n = 55)		TOTAL (%)
	Positive	%	Positive	%	
**Protists**					
*Cryptosporidium* spp.	5	7.8	3	5.4	8 (6.7)
*Cystoisospora* spp.	16	25	7	12	23 (19.3)
*Giardia intestinalis*	9	14	5	9	14 (11.7)
**Helminths**					
Ancylostomatidae	8	12.5	5	9	13 (10.9)
*Toxocara canis*	6	9.3	0	0	6 (9.3)
*Toxocara cati*	0	0	3	5.4	3 (5.4)
*Dipylidium caninum*	3	4.6	0	0	3 (4.6)
*Trichuris vulpis*	1	1.5	0	0	1 (1.5)

Of the five *Cryptosporidium* spp. positive dog fecal samples, four were from adult male animals and one was from an elderly female. As for the normality pattern, there was only one diarrheal sample. In contrast, the three cats that tested positive for *Cryptosporidium* spp. were 3-month-old males from the same domicile, and they presented a clinical condition of diarrhea.

Mixed infections by more than one gastrointestinal parasite were identified in 22.4% (13/119) of the animals, and they presented diarrheal feces (84.6%). Concomitant infection with *Cryptosporidium* spp. and *Dipylidium caninu* was observed in one dog, which presented diarrhea; unlike cats, which were infected only with *Cryptosporidium* spp.

In domiciles with presence of two or three animals, both dogs and cats, it was observed that they shared at least the same gastrointestinal parasite. The three cats infected with *Cryptosporidium* spp. belonged to the same domicile, whereas all the dogs infected with this protozoan were from different domiciles.

All samples positive for *Cryptosporidium* spp. under microscopy were amplified and sequenced. Comparison between the sequences obtained from *Cryptosporidium* isolates in dogs of the present study and the 18S rDNA sequences available in GenBank enabled identification of *C*. *parvum* in two samples (T1 and T4) and *C*. *canis* in three samples (T2, T3, and T5). In turn, the sequences obtained from isolates in cats were identified as *C*. *felis* (T6, T7, and T8).

Table 3 shows the *Cryptosporidium* species and the *C*. *parvum* subtype diagnosed in the dogs and cats assessed according to sex, age, and presence or absence of diarrhea, as well as their sequence numbers.

**Table 3:** *Cryptosporidium* spp. species and *C*. *parvum* subtype parasitizing the dogs and cats assessed according to sex, age, and presence and absence of diarrhea and the numbers of their respective sequences.

**DOG HOST**										
***Cryptosporidium* species diagnosed**	**SEX**			**AGE**			**DIARRHEA**		**Identification / Sequence number**	
	**Female**	**Male**		**≤ 1 year**	**> 1 and < 7 years**	**≥ 7 years**	**Yes**	**No**		
*Cryptosporidium parvum*	-	1		-	1	-	-	1	T1	MF589922
*Cryptosporidium parvum*	-	1		-	1	-	-	1	T4	MF589923
*Cryptosporidium canis*	-	1		-	1	-	-	1	T2	MF589918
*Cryptosporidium canis*	1	-		-	-	1	1	-	T3	
*Cryptosporidium canis*	-	1		-	1	-	-	1	T5	
**CAT HOST**										
***Cryptosporidium* species diagnosed**	**SEX**			**AGE**			**DIARRHEA**		**Identification / Sequence number**	
	**Female**		**Male**	**≤ 1 year**	**> 1 and < 7 years**	**≥ 7 years**	**Yes**	**No**		
*Cryptosporidium felis*	-		1	1	-	-	1	-	T6	MF589919
*Cryptosporidium felis*	-		1	1	-	-	1	-	T7	MF589920
*Cryptosporidium felis*	-		1	1	-	-	1	-	T8	MF589921

**Fig 3:**
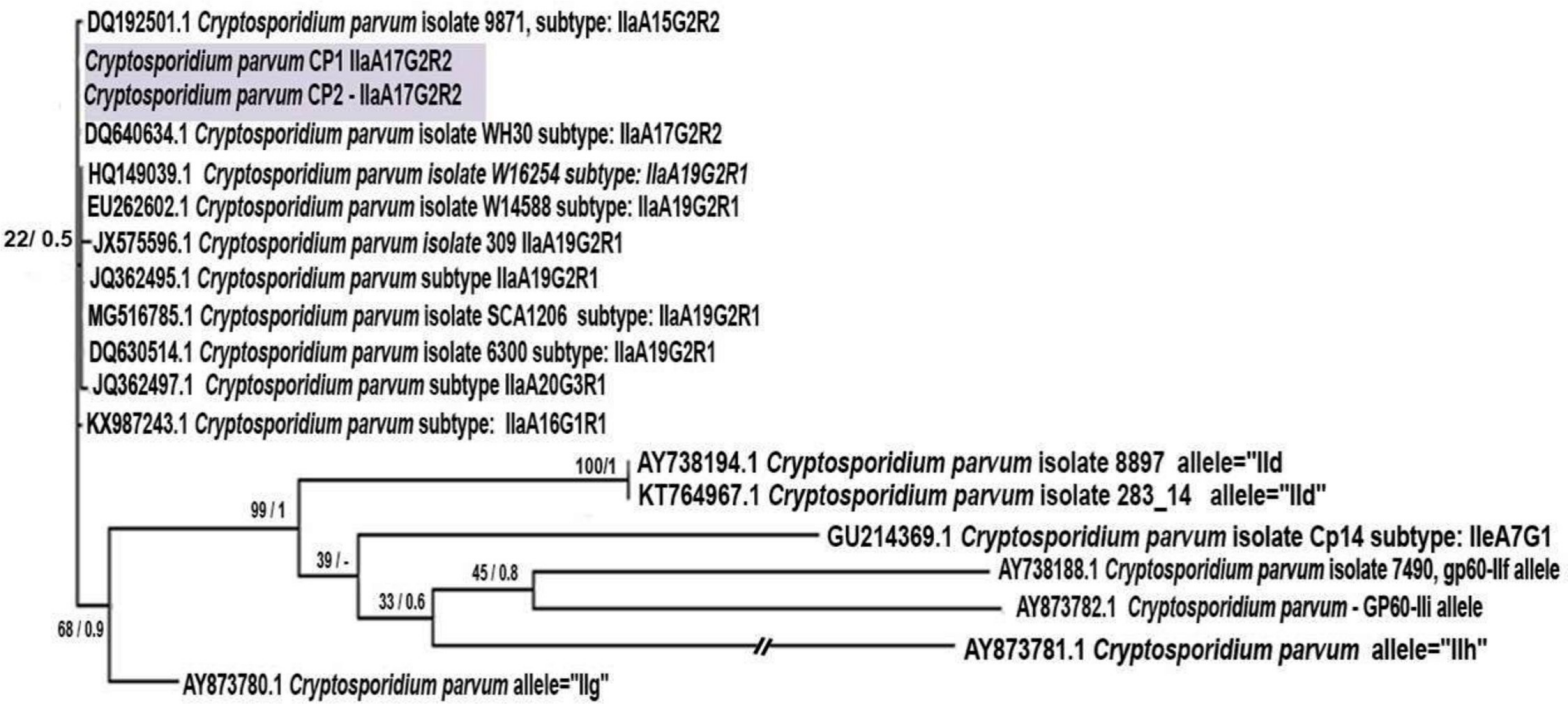
Maximum Likelihood (ML) tree of *Cryptosporidium parvum* based on GP60 partial gene using sequences obtained in the study and retrieved from Genbank. The first number associated with each node represents the ML bootstrap value followed by the Bayesian posterior probabilities. The blue rectangle represents *C*. *parvum* positive samples from this study.

Samples positive for *C*. *parvum* (T1 and T4) were identified as subtype IIaA17G2R2 after analysis of the GP60 locus. The sequence was deposited in GenBank under accession number MH715474. Phylogenetic analysis of the *C*. *parvum* subtype sequences showed that they were associated with the subtype IIa family (Fig 3).

## Discussion

The low prevalence rate found in the present study is consistent with the findings of some previous surveys conducted with dogs and cats also domiciled in Brazil [24, 29, 30, 34], of studies conducted in other countries, such as Germany and Greece, having cats as host [6, 52], and of a research conducted with dogs in Japan [53].

Nevertheless, higher [54] and lower [6, 19] percentages compared with that of this study have also been found. However, when comparing different surveys, some factors that may influence infection rate, such as sample size, should be considered [55]. There is a report of a relatively high percentage (41.67%), with five of the 12 investigated cats infected with *Cryptosporidium* [54]. In this case, a question remains as to whether this result represents the local reality or the sample size was small. Other aspects that can influence this result are the methodology used, regional variables, and homogeneity of the target population. It is worth noting that *Cryptosporidium* spp. oocysts are eliminated by the host intermittently, both in symptomatic and asymptomatic individuals. Thus, many fecal samples should be evaluated before a final diagnosis is made [29, 56]. Based on this, the larger the number of samples per individual, the greater the chances of obtaining a positive diagnosis of *Cryptosporidium* spp., especially in environments where there is infection [5]. In the present study, collections of single samples were made because of some limitations, such as difficulty in finding owners of dogs and cats who agreed to participate in the research and the non-compliance with the procedures by some participants. Thus, the infection rates presented may have been underestimated.

Only one of the five dogs infected with *Cryptosporidium* spp. showed clinical signs of diarrhea. This result corroborates the view that this infection is generally asymptomatic in dogs [8, 57, 58]. In the case of cats, symptoms often are also absent [8, 31]; however, in this study, all infected cats presented diarrheal feces. The explanation for this may lie in the age of the animals analyzed, as infections in young animals regularly develop along with diarrhea [35].

Although the population of cats in this study was predominantly composed of adult animals (58.2%), three of the cats infected with *Cryptosporidium* spp. were only three months old. Young cats seem to be more susceptible to infections, probably because their immune system is still developing and, therefore, these animals have not yet achieved full immunity, which is acquired through previous exposure to parasites [59, 60]. Higher *Cryptosporidium* spp. prevalence rates in young cats have also been identified in previous studies [8, 21, 35].

In this work, almost all dogs infected with *Cryptosporidium spp*. were adults. A previous study [20] reported a higher frequency of infection in adult dogs (10.1%) compared with that in young animals (5.5%); however, there are reports of infection in any age group [11, 22, 53].

Although these were companion animals, most of them had access to the external area of their domiciles, as well as to their neighboring domiciles, and were thus more exposed to sources of infection and to contact with other infected animals of the same or different species. In addition, they may have acquired infection through environmental contamination by oocysts brought by rainwater runoff, or through ingestion of water and food unsuitable for consumption. It is a well-known fact that *Cryptosporidium* spp. oocysts are extremely resistant to environmental adversities, spread easily through water, and are resistant to conventional water treatments, such as chlorination and filtration [61, 62]. In addition, ingestion of possibly contaminated raw or undercooked foods can be a risk factor for *Cryptosporidium* spp. infection [27, 59].

Corroborating the findings of other surveys [8, 11], the species *C*. *canis* and *C*. *felis* were diagnosed, respectively, in the dogs and cats assessed in this study, indicating that *C*. *canis* seems to present a certain degree of specificity in dogs, while *C*. *felis* has it in cats.

In Brazil, molecular characterization of *Cryptosporidium* spp. in dogs and cats is still little explored [23, 28, 36]. The aforementioned authors have reported identification of *C*. *canis* in dogs and *C*. *felis* in cats. In this study, *C*. *parvum* was identified in fecal samples of dogs. This species has occasionally been reported in both dogs and cats [6, 12, 13, 63, 64].

Due to the presence of *C*. *parvum* in the study sample, there is a significant and imminent risk of zoonotic transmission of *Cryptosporidium* between the owners and their companion dogs. Nevertheless, although *C*. *canis* and *C*. *felis* are more limited to zoonotic transmissions, they also deserve attention, especially regarding more vulnerable humans exposed to risk of infections. In addition, the possible implications for the health of dogs and cats infected by these species cannot be disregarded. Due to the impact of these species on health, further research should be conducted in order to better understand the zoonotic transmission potential of *Cryptosporidium* spp. between humans and their pets, specifically dogs and cats.

Sequence analyses using the GP60 gene for *C*. *parvum* are important because not all its subtypes present zoonotic potential. Families of subtypes are found in this species, and the subtype IIa has already been diagnosed in humans and ruminants, being responsible for zoonotic cryptosporidiosis. However, the *C*. *parvum* subtypes IIaA17G1R1 and IIaA15G2R1 have previously been identified in dog fecal samples [65].

The subtype described in this study (IIaA17G2R2), diagnosed in two dogs, has already been diagnosed in humans [66, 67]. This finding suggests that zoonotic transmission may occur in the study region, but opens space for future questions: Has this subtype been adapted to dogs? Did this subtype already exist in dogs? Had it not yet been diagnosed in this host? Even because the samples were collected in a region with peri-urban characteristics with presence of production animals in its surroundings. Therefore, the possibility of environmental contamination with bovine and/or human feces containing *C*. *parvum* oocysts of the subtype diagnosed in dogs in this study cannot be discarded. Thus, the dogs could have been infected through ingestion of contaminated water and/or food and become new possible hosts of this subtype that, until then, was commonly diagnosed in cattle and humans.

In most cases, molecular detection of *Cryptosporidium* spp. in fecal samples is only possible trough two-step PCR (nested PCR) followed by genetic sequencing, which are the most widely used combined techniques that allow classification of all *Cryptosporidium* species and genotypes, thus providing subsidies to investigate probable sources and routes of transmission. In addition to the three species of *Cryptosporidium*, virtually all other gastrointestinal parasites diagnosed, except for *Cystoisospora* spp. and *Trichuris vulpis* (Table 2), are agents of zoonotic diseases considered relevant to public health [24]. The identification of these parasites serves as an alert for the study area, and they should not be neglected, considering that application of preventive measures can reduce the risk of transmission to humans and animals.

## Conclusions

Some of the cats and dogs investigated in this study were positive for *Cryptosporidium* spp., with both symptomatic and asymptomatic hosts. Two *Cryptosporidium* species (*Cryptosporidium parvum* and *Cryptosporidium canis*) were observed in the local canine population, whereas only the species *C*. *felis* was identified in the feline population. The three species diagnosed, in addition to being debilitating for their hosts, present zoonotic potential. With respect to the canine host, further research is needed to trace the molecular epidemiology of this etiologic agent, considering that a subtype of a family with zoonotic potential was diagnosed in the *C*. *parvum* species in the present study.

## Supporting information

S1 File *Cryptosporidium* species and zoonotic subtype identified in this study. (DOCX)
