# Supplementary material for: Molecular characterization of Cryptosporidium spp. in dogs and cats in the city of Rio de Janeiro, Brazil, reveals potentially zoonotic species and genotype
Source: PLoS One. 2021 Aug 3;16(8):e0255087. doi: 10.1371/journal.pone.0255087 (PMC8330930; doi:10.1371/journal.pone.0255087)
Supplement: S1 File — (DOCX) [file pone.0255087.s001.docx]

| **Species / sample / accession number** | **Identity**  **(%)** | **Sequence** |
| --- | --- | --- |
| ***Cryptosporidium parvum*** | | |
| **Sample T1**  MF589922 | 100 – 99 | 1 tcatatactt tacggatcac attaaatgtg acatatcatt caagtttctg acctatcagc  61 tttagacggt agggtattgg cctaccgtgg caatgacggg taacggggaa ttagggttcg  121 attccggaga gggagcctga gaaacggcta ccacatctaa ggaaggcagc aggcgcgcaa  181 attacccaat cctaatacag ggaggtagtg acaagaaata acaatacagg actttttggt  241 tttgtaattg gaatgagtta agtataaacc cctttacaag tatcaattgg agggcaagtc  301 tggtgccagc agccgcggta attccagctc caatagcgta tattaaagtt gttgcagtta  361 aaaagctcgt agttggattt ctgttaataa tttatataaa atattttgat gaatatttat  421 ataatattaa cataattcat attactatat attttagtat atgaaatttt actttgagaa  481 aattagagtg cttaaagcag gcatatgcct tgaatactcc agcatggaat aatattaaag  541 atttttatct ttcttattgg ttctaagata agaataatga ttaataggga cagttggggg  601 catttgtatt taacagtcag aggtgaaatt cttagatttg ttaaagacaa actaatgcga  661 aagcatttgc caaggatgtt ttcattaatc aagaacgaaa gttaggggat cgaagacgat  721 cagataccgt cgtagtc |
|  |  |  |
|  |  |  |
|  |  |  |
| **Sample T4**  MF589923 | 100 | 1 tcataataac tttacggatc acattaaatg tgacatatca ttcaagtttc tgacctatca  61 gctttagacg gtagggtatt ggcctaccgt ggcaatgacg ggtaacgggg aattagggtt  121 cgattccgga gagggagcct gagaaacggc taccacatct aaggaaggca gcaggcgcgc  181 aaattaccca atcctaatac agggaggtag tgacaagaaa taacaataca ggactttttg  241 gttttgtaat tggaatgagt taagtataaa cccctttaca agtatcaatt ggagggcaag  301 tctggtgcca gcagccgcgg taattccagc tccaatagcg tatattaaag ttgttgcagt  361 taaaaagctc gtagttggat ttctgttaat aatttatata aaatattttg aatatttata  421 taacattaac ataattcata ttactatatt ttttagtata tgaaatttta ctttgagaaa  481 attagagtgc ttaaagcagg catatgcctt gaatactcca gcatggaata atattaaaga  541 tttttatctt tcttattggt tctaagataa gaataatgat taatagggac agttgggggc  601 atttgtattt aacagtcaga ggtgaaattc ttagatttgt taaagacaaa ctaatgcgaa  661 agcatttgcc aaggatgttt tcattaatca agaacgaaag ttaggggatc gaagacgatc  721 agataccgtc gtagtc |
|  |  |  |
|  |  |  |
|  |  |  |
| ***Cryptosporidium canis*** | | |
| **Samples**  **T2, T3 and T5**  MF589918 | 100 | 1 tgattcataa taactttacg gatcacattt tatgtgacat atcattcaag tttctgacct  61 atcagcttta gacggtaggg tattggccta ccgtggcaat gacgggtaac ggggaattag  121 ggttcgattc cggagaggga gcctgagaaa cggctaccac atctaaggaa ggcagcaggc  181 gcgcaaatta cccaatccta atacagggag gtagtgacaa gaaataacaa tacaggactt  241 taacagtttt gtaattggaa tgagttgagt ataaacccct ttacaagtat caattggagg  301 gcaagtctgg tgccagcagc cgcggtaatt ccagctccaa tagcgtatat taaagttgtt  361 gcagttaaaa agctcgtagt tggatttctg ttaataattt atatataata tttaacatat  421 ttatataata ttaacataat tcatattact atttatagta tatgaaactt tactttgaga  481 aaattagagt gcttaaagca ggcttttgcc ttgaatacta gagcatggaa taatattaaa  541 gatttttatc tttcttattg gttctaagat agaaataatg attaataggg acagttgggg  601 gcatttgtat ttaacagtta gaggtgaaat tcttagattt gttaaagaca aactaatgcg  661 aaagcatttg ccaaggatgt tttcattaat caagaacgaa agttagggga tcgaagacga  721 tcagataccg tcgtagtct |

**Table 1. *Cryptosporidium* species from dogs e cats obtained in the present study.**

| ***Cryptosporidium felis*** | | |
| --- | --- | --- |
| **Sample T6**  MF589919 | 98 - 99 | 1 tcagctttag acggtagggt attggcctac cgkggctatg acgggtaacg gggaattagg  61 gttcgattcc ggagagggag cctgagaaac ggctaccaca tctaaggaag gcagcaggcg  121 cgcaaattac ccaatcctaa tacagggagg tagkgacaag aaataacaat acaggacttt  181 acggttttgt aattggaatg agttaagtat aaaccccttt acaagtatca attggagggc  241 aagtctggkg ccagcagccg cggtaattcc agctccaata gcgtatatta aagttgttgc  301 agttaaaaag ctcgwagttg gatttctgtt aataccttat atataatatt tttttttaaa  361 wwttatwatg kaaaattaac awaattcawa ttttttaaga ctgaattttt agttttgata  421 atatgaaatt ttactttgag aaaattagag tgcttaaagc aggcttttgc cttgaatact  481 ccagcatgga ataataataa aagattttta tctttttttt attggttcta agataaaaat  541 aatgattaat agggacagtt gggggcattt gtatttaaca gtcagaggtg atattcttag  601 atttgttaaa gacaaactaa tgcgaaagca tttgccaagg atgttttcat taatcaagaa  661 cgaaagttag gggatcgaag acgatcagat ac |
| **Sample T8**  MF589921 |  | 1 tcagctttag acggtagggt attggcctac cgkggctatg acgggtaacg gggaattagg  61 gttcgattcc ggagagggag cctgagaaac ggctaccaca tctaaggaag gcagcaggcg  121 cgcaaattac ccaatcctaa tacagggagg tagtgacaag aaataacaat acaggacttt  181 acggttttgt aattggaatg agttaagtat aaaccccttt acaagtatca attggagggc  241 aagtctggtg ccagcagccg cggtaattcc agctccaata gcgtatatta aagttgttgc  301 agttaaaaag ctcgwagttg gatttctgtt aataccttat atataatatt tttttttaaa  361 tattwttatg taagattaac ataattcata ttttttaaga ctgaattttt agttttgata  421 atatgaaatt ttactttgag aaaattagag tgcttaaagc aggcttttgc cttgaatact  481 ccagcatgga ataataataa aagattttta tctttttttt attggttcta agataaaaat  541 aatgattaat agggacagtt gggggcattt gtatttaaca gtcagaggtg atattcttag  601 atttgttaaa gacaaactaa tgcgaaagca tttgccaagg atgttttcat taatcaagaa  661 cgaaagttag gggatcgaag acgatcagat ac |
| **Sample 17**  MF589920 | 100 | 1 tcagctttag acggtagggt attggcctac cgtggctatg acgggtaacg gggaattagg  61 gttcgattcc ggagagggag cctgagaaac ggctaccaca tctaaggaag gcagcaggcg  121 cgcaaattac ccaatcctaa tacagggagg tagtgacaag aaataacaat acaggacttt  181 acggttttgt aattggaatg agttaagtat aaaccccttt acaagtatca attggagggc  241 aagtctggtg ccagcagccg cggtaattcc agctccaata gcgtatatta aagttgttgc  301 agttaaaaag ctcgtagttg gatttctgtt aataccttat atataatatt tttttttaaa  361 tattattatg taagattaac ataattcata ttttttaaga ctgaattttt agttttgata  421 atatgaaatt ttactttgag aaaattagag tgcttaaagc aggcttttgc cttgaatact  481 ccagcatgga ataataataa aagattttta tctttttttt attggttcta agataaaaat  541 aatgattaat agggacagtt gggggcattt gtatttaaca gtcagaggtg atattcttag  601 atttgttaaa gacaaactaa tgcgaaagca tttgccaagg atgttttcat taatcaagaa  661 cgaaagttag gggatcgaag acgatcagat ac |
|  |  |  |
|  |  |  |

**Table 2. *Cryptosporidium parvum* subtype obtained in the present study.**

| *C. parvum* subtype/ **sample / accession number** | **Identity**  **(%)** | **Sequence** |
| --- | --- | --- |
| **Sample**  T1 and T4  MH715474 | **100** | 1 tcatcatcgt catcgtcatc atcatcatca tcatcatcat catcatcatc atcatcaaca  61 tcaacatcaa ccgtcgcacc agcaaataag gcaagaactg gagaagacgc agaaggcagt  121 caagattcta gtggtactga agcttctggt agccagggtt ctgaagagga aggtagtgaa  181 gacgatggcc aaactagtgc tgcttcccaa cccactactc cagctcaaag tgaaggcgca  241 actaccgaaa ccatagaagc tactccaaaa gaagaatgcg gcacttcatt tgtaatgtgg  301 ttcggagaag gtaccccagc tgcgacattg aagtgtggtg cctacactat cgtctatgca  361 cctataaaag accaaacaga tcccgcacca agatatatct ctggtgaagt tacatctgta  421 acctttgaaa agagtgataa tacagttaaa atcaaggtta acggtcagga tttcagcact  481 ctctctgcta attcaagtag tccaactgaa aatggcggat ctgcgggtca ggcttcatca  541 agatcaagaa gatcactctc agaggaaacc agtgaagctg ctgcaaccgt cgatttgttt  601 gcctttaccc ttgatggtgg taaaagaatt gaagtggctg taccaaacgt cgaagatgca  661 tctaaaagag acaagtacag tttggttgca gacgataaac ctttctatac cggcgcaaac  721 agcg |
